# First co-infection case of melioidosis and Japanese encephalitis in China

**DOI:** 10.1186/s12879-018-3364-6

**Published:** 2018-09-04

**Authors:** X. Y. Li, B. X. Ke, C. N. Chen, H. L. Xiao, M. Z. Liu, Y. C. Xiong, R. Bai, J. D. Chen, C. W. Ke

**Affiliations:** 1 School of Public Health, Southern Medical University, No.1023 Shatainan Road, Baiyun District, Guangzhou, 510515 Guangdong Province China; 2 Institute of Pathogenic Microbiology, Guangdong Provincial Center for Disease Control and Prevention, No.160 Qunxian Road, Panyu District, Guangzhou, 511430 Guangdong Province China; 3 Tonghu Hospital, ZhongKai high-tech District, Huizhou, 516000 Guangdong Province China; 4 Huizhou City Center for Disease Control and Prevention, No.10 Fumin Road Huicheng District, Huizhou, 516000 Guangdong Province China

**Keywords:** *Burkholderia pseudomallei*, JEV, Co-infection, First case, Central nervous system infection

## Abstract

**Background:**

Melioidosis is endemic in Southeast Asia and northern Australia. Infection usually follows percutaneous inoculation or inhalation or ingestion of the causative bacterium, *Burkholderia pseudomallei*, which is present in soil and surface water in endemic regions. Japanese encephalitis (JE) is a vector-borne viral zoonosis caused by Japanese encephalitis virus (JEV), leading to epidemic encephalitis in Southeast Asia. Both *B. pseudomallei* and JEV have spread dominantly in the Hainan and Guangdong provinces in China. Here we reported the first case of co-infection of *B. pseudomallei* and JEV, which was discovered in Huizhou in the Guangdong province in June 2016.

**Case presentation:**

A 52-year-old man was admitted to the hospital with acute febrile illness and headache, diagnosed as respiratory infection, central nervous system (CNS) infection, septicemia, and hepatic dysfunction. Based on *B. pseudomallei*-positive blood and cerebrospinal fluid (CSF) cultures, the patient was diagnosed with melioidosis and treated aggressively with antibiotics. However, the patient failed to make a full recovery. Further laboratory tests focused on CNS infection were conducted. The co-infection of *B. pseudomallei* and JEV was confirmed after the positive IgM antibodies of JEV were detected in both CSF and blood. After diagnosis of co-infection with *B. pseudomallei* and JEV, the patient was provided supportive care in hospital and recovered after approximately 3 weeks.

**Conclusion:**

Given the possibility of co-infection of *B. pseudomallei* and JEV, as well as variable case presentations, it is critical to enhance the awareness, detection, and treatment of co-infection in regard to melioidosis.

## Background

*Burkholderia pseudomallei* is an ancient and often neglected bacterium, causes melioidosis. The disease endemic in subtropical regions between latitudes 20°N and 20°S, especially in Northern Australia and Southeast Asia [1]. The routes of infection include inhalation, aspiration, ingestion, and percutaneous inoculation [2, 3]. It is very rare for this pathogen to be transmitted from person-to-person [4]. The clinical manifestations of melioidosis are varied and present as pneumonia, septicemia, single, or multiple abscess. The geographical distribution of *B. pseudomallei* in China is characterized by the climates, and positive isolation mainly occurs in southern areas like Guangdong, Hainan and Guangxi where the temperature in January is above 12 °C [5], and cases of melioidosis are usually published as case report. The study conducted by Jiang ZJ [6] reported that *B. pseudomallei* was discovered in water samples collected from Huizhou, Guangdong province. Furthermore, it was also detected in some patients whose serum showed positive for an antibody against *B. pseudomallei* in Guangdong with the positive rate was 6.1% in 1988 [5]. Subsequently, sporadic melioidosis cases have been reported in Hainan province and Zhanjiang, Guangdong province [7, 8], with a recent clinical study in Hainan showing an increased incidence of melioidosis being reported in this region [9]. However, cases were rarely reported in Guangdong province over the most recent 5 years, which may result from lacking of recognition and underreporting .

Japanese encephalitis is the most important mosquito-borne viral infectious disease in parts of Southeast Asia. The disease is caused by Japanese encephalitis virus and is typically an acute neurological syndrome, characterized by fever, convulsions, headache, focal neurological sign, and decreased consciousness. Diseases caused by Japanese encephalitis viruses and by *B. pseudomallei* are similar, but they are treated very differently. It has been unusual to detect *B. pseudomallei* with other pathogens in patients. Dengue and melioidosis coinfection was reported in Northeastern Brazil [10]; A case of leptospirosis and melioidosis co-infection occurred in Malaysia [11]. Melioidosis coinfection with other disease e.g. cutaneous leishmaniasis, pulmonary tuberculosis and cryptococcosis also has been reported in different countries [12–14]. In this study, we reported the first case that was diagnosed with both Japanese encephalitis and Melioidosis.

## Case presentation

A 52-year-old male without a history of hypertension and diabetes, from Huizhou, Guangdong, was referred to Huizhou Central People’s Hospital in June 2016 with symptoms of fever for 1 week. He worked on a farm with poor hygiene management and was responsible for breeding. Before admission, he was treated with flu in the local community clinic, but respiratory infection, CNS infection, septicemia, and hepatic dysfunction were suspected when he was admitted to the Central People’s Hospital.

During physical examination, he had fever (38.1°C) and headache with normal blood pressure (100/80 mmHg). All respiratory, cardiovascular and neurological examinations were normal. There was no evident sign of neck stiffness, initial unilateral limb weakness or flaccid paraparesis. Hepatosplenomegaly and peripheral edema were also not observed.

The initial laboratory investigation displayed high white blood cell (WBC) count of 10.2 × 10^^9^/L with predominant 88.4% neutrophil, normal hemoglobin and platelet count. The liver profile showed elevated total bilirubin (TBIL) 39.7umol/L, direct bilirubin(DBIL)19.9umol/L, alanine aminotransferase (ALT)75 U/L, and aspartate transaminase(AST)46 U/L; renal function was normal. The chest computed tomography showed slight bilateral fiber tissue hyperplasia. These examinations strongly suggested typical infection by bacteria with a variety of clinical manifestations.

A lumbar puncture was conducted, and a CSF biochemical test was subsequently performed. The WBC count was 72.0 × 10^6/L, showing predominantly mononuclear cells, and the cerebrospinal fluid pressure (CSFP) was 220 mmH_2_O. The C-reactive protein was 139.87 mg/L. The level of protein in CSF was elevated, while that of glucose was normal. To confirm the pathogen leading to infection, both the blood and CSF were cultured for further characterization, as described below. Moreover, bilateral brain parenchymal enhancement of lesions with an obvious enhancement of left lateral pterygoid and temporalis were observed in the following brain Magnetic Resonance Imaging (MRI). The Magnetic Resonance Angiography (MRA) of the cerebral arteries was normal. Furthermore, testing was all negative for tuberculosis, autoimmune disease, acid-fast bacilli, Cryptococcus, and Hepatitis B virus. Combined with the above results, the patient was diagnosed with neurological melioidosis (Table 1). It took a week to diagnose this patient in the hospital.

**Table 1:** The biochemical and immunological test results of the patient

Samples	Test items	June					
		18th	19th	21th	22th	25th	29th
Blood	WBC Count/(×10^9/L)	12.1 (↑)	10.2 (↑)				
	Neutrophil/(%)		88.4 (↑)				
	TBIL/(μmol/L)	39.7 (↑)					
	ALT/(U/L)	75 (↑)					
	AST/(U/L)	46 (↑)					
	ANA test			(−)			
	Culture/*B. pseudomallei*					(+)	
	JEV IgM						(+)
CSF	WBC Count/(×10^9/L)			72 (↑)			
	C-reactive protein(mg/L)				139.87 (↑)		
	acid-fastbacilli				(−)		
	*Cryptococcus neoformans*				(−)		
	Culture/*B. pseudomallei*					(+)	
	JEV IgM						(+)

(+), positive; (−), negative; (↑), higher than the standard value

**Fig. 1:**
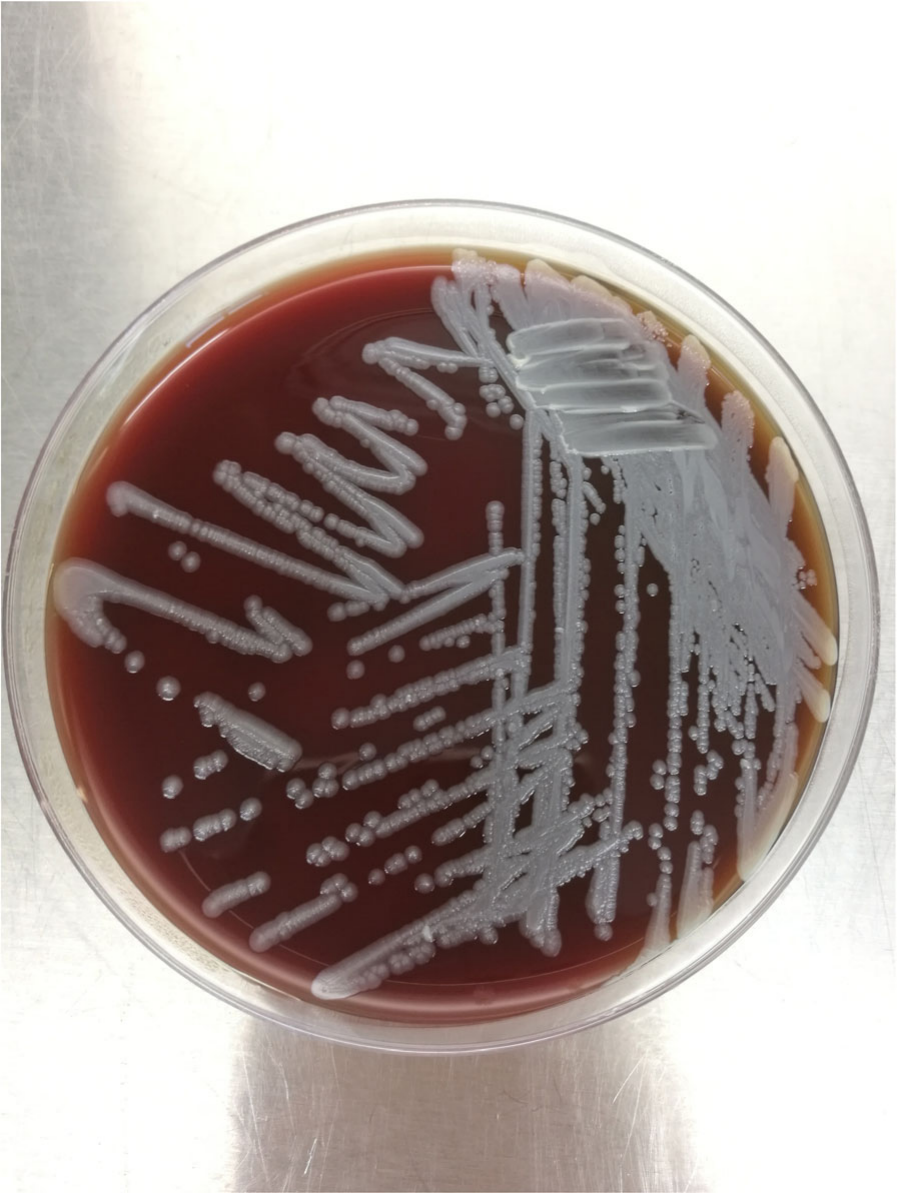
The isolation of *B. pseudomallei* in blood plate

**Fig. 2:**
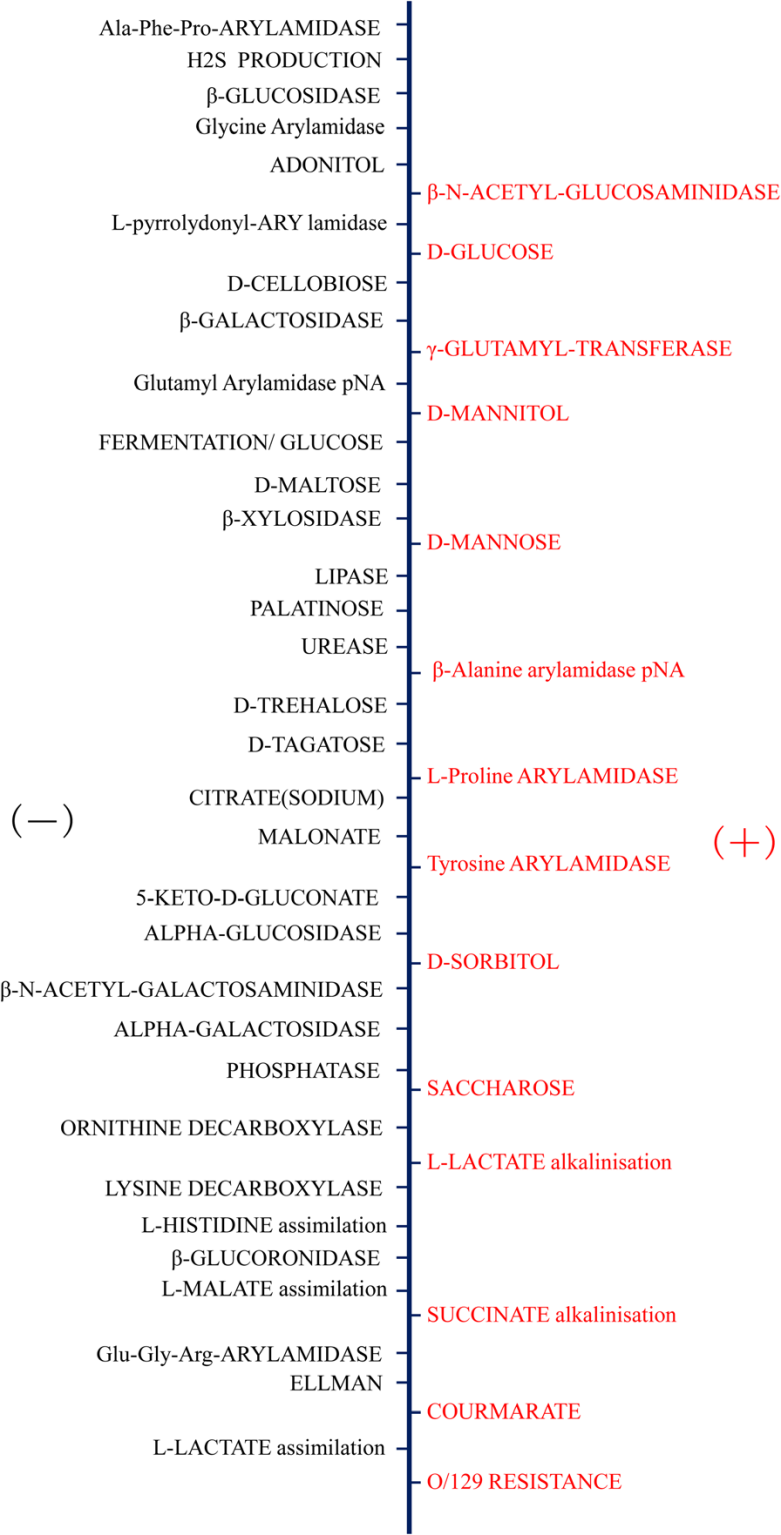
The detail results of biochemical detection. (+) side represents positive item, (−)side represents negative item

Despite the patient being treated with intravenous injection of ceftazidime at a dose of 2 g 12-hourly, The patient became agitated, and an intermittently persistent fever was observed during treatment of this patient. Samples of the patient’s blood and CSF were sent to the Guangdong provincial CDC (No.160 Qunxian Road, Panyu District, Guangzhou, Guangdong province, China) for further identification of CNS infection. The culture results coincided with those in hospital, excluding other bacterial and fungal infections. Both sample were cultured on blood plate. After incubating the blood and CSF specimens at 35°C on blood agar for 48 h, a single grey wheel-shaped isolates morphology was observed (Fig. 1). This culture was tested with biochemical and molecular diagnostic method to identify the pathogen. The isolates were tested utilizing the Automatic microbiological analysis system(VITEK 2 Systems, Biomerieux, France), the biochemical results details listing in the Fig. 2, and *B. pseudomallei* was identified with 99% probability. The DNA of the isolates, CFS and blood were extracted (QiAamp DNA kit) and real-time PCR (*B. pseudomallei* real-time PCR kit, shanghai ZJ bio-tech, China) results were positive for *B. pseudomallei*, confirming the original clinical diagnosis of neurological melioidosis. The presence of IgM antibodies specific to JEV were detected using ELISA(JE Detect IgM ELISA, InBios, America) and were confirmed as positive, revealing the *B. pseudomallei* and JEV co-infection in this patient. Considering the possibility of cross-reacting with DENV, the serum sample was detected for specific antibodies against DENV and result showed negative(DENGUE DUO CASSETTE, Panbio, Australia). Furthermore, the detection was negative for screening viruses including enterovirus, influenza virus, adenovirus, and poliovirus by ELISA. The patient received symptomatic management with ceftazidime (2 g 12-hourly), oxiracetam (6 g per day) and dipyridamole (150 mg per day). After 22 days of treatment, he was discharged on eradication therapy with oral 960 mg trimethoprim-sulfamethoxazole (160 mg trimethoprim and 800 mg sulfamethoxazole) per 12 h for 2 months. At follow-up 3 months after initial admission, the patient had recovered well without fever or headache. There was no growth of *B. pseudomallei* on CSF culture in physical review from Aug.

## Discussion

Guangdong province is located in a subtropical area with abundant rainfall, which offers suitable conditions for the existence of *B. pseudomallei*. *B. pseudomallei* has been recognized for more than 100 years since 1911, when it was first reported in Rangoon. The first case report in China occurred in Hong Kong in 1984, and the following case occurred in Taiwan [15, 16]. Since then, sporadic cases have been reported in different districts of the mainland, especially in Hainan and Guangdong [17–19]. Over the most recent 5 years, however, almost no cases have been reported in Guangdong. Huizhou may also be deemed an endemic area even though no human cases have been reported until this report. Because of the complicated manifestations of the disease and weak awareness among the population, the morbidity of melioidosis has likely been underestimated.

Melioidosis is associated with exposure to the soil and water, and the patients lived on a farm with poor hygiene. Like other melioidosis-endemic regions, recent heavy rainfall may increase the risk of melioidosis in tropical Chinese provinces. The patient described in our study lived in a region that had experienced a week of heavy rainfall prior to their symptom onset, increasing opportunities for *B. pseudomallei* exposure; e.g., inhalation and direct skin inoculation, since the pathogen tends to be aerosolized and the amount of bacteria in the water is increased. These are several risk factors for acquiring the disease, whereas there was no history of travel to other endemic areas, which suggests this was a local case.

The patient was most likely co-infected with JEV, a prevalent disease occurring during the summer and autumn, especially among children aged 1 to 14, yet there has been an increased incidence among ≥40 years old cases in recent years in China [20]. This patient is over 40 years old, which reminds us that underlying diseases and low immune levels are two risk factors for adult cases. Improving the vaccination rate is the most effective strategy for JE control and prevention. Government authorities should expand the scope of the vaccination age range. Moreover, the sequence of patient infecting melioidosis and JE has not yet been concretely established. Maybe the JEV infection is a risk factor for melioidosis like diabetes, heavy alcohol consumption and malignancy. Conversely, maybe the infection of *B. pseudomallei* was antecedent to that of JEV, or it was a simultaneous infection.

Neurological melioidosis is unusual, with the proportion representing as little as 5% of overall melioidosis [21]. Therefore, it is commonly overlooked and misdiagnosed. Reasonable diagnosis and proper treatment will also be of vital importance to a good outcome. In our case, we used different tools to identify the infection of *B. pseudomallei* including culture, biochemical test and PCR, which all obtained the same result, with a series discrimination experiments pinning the JEV infection. Since this case represented a mild infection, the clinical manifestations of the patient were not typical, just with fever, headache and malaise, and the symptoms were improved with our intervention. *B. pseudomallei* is commonly susceptible to ceftazidime, amoxycillin-clavulanic acid, penicillin, imipenem, azlocillin, ticarcillin-vulanic acid, aztreonam and ceftriaxone but resistant to gentamicin and colistin. But now the chose of antibiotics is more mature and most melioidosis cases can easily be treated with suitable therapies [22]. Ceftazidime is an important therapeutic option treatment along with other antibiotics. Our patient had intravenous ceftazidime for 3 weeks, in combination with oral maintenance therapy (trimethoprim and sulfamethoxazole) for 2 month and recovered well.

Given the history of Guangdong serving as an epidemic area, and the patient’s clinical features, we tested our patient for JEV on the twelfth day, and JEV IgM was positive in CSF. The confirmation time can be earlier when enough attention is paid. Worldwide, approximately 35,000–50,000 people suffer from JE every year, with a mortality rate of 10,000–15,000 people per year [23]. Delay in diagnosis may be a factor contributing to this high mortality.

## Conclusion

Guangdong is an endemic area for melioidosis, and considering the prevalence of similar situations in China, we have to focus on the rural patients in our country. To our knowledge, this is the first reported case of co-infection of melioidosis and JE. With climate change and the severe situation of antibiotic-resistant bacteria, physicians should strengthen awareness of neurological melioidosis, JE and the multiple co-infections in Guangdong, China.

## Abbreviations

ALT: Alanine aminotransferase
AST: Aspartate transaminase
CNS: Central nervous system
CSF: Cerebrospinal fluid
CSFP: Cerebrospinal fluid pressure
DBIL: Direct bilirubin
JE: Japanese encephalitis
JEV: Japanese encephalitis virus
TBIL: Total bilirubin
WBC: White blood cell
